# Au Nanocage Functionalized with Ultra-small Fe_3_O_4_ Nanoparticles for Targeting *T*_*1*_–*T*_*2*_Dual MRI and CT Imaging of Tumor

**DOI:** 10.1038/srep28258

**Published:** 2016-06-17

**Authors:** Guannan Wang, Wei Gao, Xuanjun Zhang, Xifan Mei

**Affiliations:** 1 College of Pharmacy & the Key Laboratory for Medical Tissue Engineering of Liaoning Province, Liaoning Medical University, Jinzhou, 121001, China; 2 Faculty of Health Sciences, University of Macau, Avenida da Universidade, Taipa, Macau, China

## Abstract

Diagnostic approaches based on multimodal imaging of clinical noninvasive imaging (eg. MRI/CT scanner) are highly developed in recent years for accurate selection of the therapeutic regimens in critical diseases. Therefore, it is highly demanded in the development of appropriate all-in-one multimodal contrast agents (MCAs) for the MRI/CT multimodal imaging. Here a novel ideal MCAs (F-AuNC@Fe_3_O_4_) were engineered by assemble Au nanocages (Au NC) and ultra-small iron oxide nanoparticles (Fe_3_O_4_) for simultaneous *T*_*1*_–*T*_*2*_dual MRI and CT contrast imaging. In this system, the Au nanocages offer facile thiol modification and strong X-ray attenuation property for CT imaging. The ultra-small Fe_3_O_4_ nanoparticles, as excellent contrast agent, is able to provide great enhanced signal of *T*_*1*_- and *T*_*2*_-weighted MRI (*r*_*1*_ = 6.263 mM^−1^ s^−1^, *r*_*2*_ = 28.117 mM^−1^ s^−1^) due to their ultra-refined size. After functionalization, the present MCAs nanoparticles exhibited small average size, low aggregation and excellent biocompatible. *In vitro and In vivo* studies revealed that the MCAs show long-term circulation time, renal clearance properties and outstanding capability of selective accumulation in tumor tissues for simultaneous CT imaging and *T*_*1*_- and *T*_*2*_-weighted MRI. Taken together, these results show that as-prepared MCAs are excellent candidates as MRI/CT multimodal imaging contrast agents.

The development of personalized therapeutical approaches and the increasing precision of surgical techniques indicate the importance of multimodal imaging to assist physicians in diagnosis and monitoring the response to therapy. In particular, noninvasive imaging (eg. MRI and CT) and minimally invasive *in vivo* bioimaging techniques are valuable tools in the arsenal of clinical diagnostics^1^,^2^,^3^. The new emergence of MRI/CT scanner (eg. General Electric CT & MRI scanners) allows doctors to get more precise information of tumor localization and boundary identification by combination of MRI and CT imaging. Accompanied with the development of imaging technology, high-performance, especially those all-in-one multimodal contrast agents (MCAs) are highly demanded for accurate diagnosis and therapy. In the past several years, various of MCAs based on Au and Fe_3_O_4_ nanoparticles have been developed for *in vivo* and pre-clinical MRI/CT imaging with the purpose of increasing the contrast of lesion, because these nanoparticles can offer facile thiol modification, enhanced chemical stability, excellent biocompatibility, superparamagnetic capability, and strong X-ray attenuation property^4^,^5^,^6^,^7^,^8^,^9^,^10^,^11^,^12^. However, the achievement of these MCAs by using Fe_3_O_4_ and Au shell always have low MRI contrast capability, because common Fe_3_O_4_ can only provide *T*_*2*_ weight MRI with dark imaging and Au shell coated on the surface of Fe_3_O_4_ also prevent the connection with water molecules in tissue resulting in the reduction of the MRI contrast signal.

To address above problem, here we have exploited a new folic acid functionalized MCAs (F-AuNC@Fe_3_O_4_), based on Au nanocages (AuNCs) and ultrasmall Fe_3_O_4_ nanoparticles, to associate in one signal nanosystem several different properties, such as, tumor targeting, bright *T*_*1*_ and dark *T*_*2*_dual MRI contrast enhancement, and X-ray attenuation (Fig. 1). Compared with other multimodal imaging systems, our presented MCAs are the first engineered for simultaneous *T*_*1*_–*T*_*2*_dual MRI and CT contrast. The simultaneous *T*_*1*_ and *T*_*2*_ weight contrast imaging could great enhance the sensitivity of MRI to give the comprehensive high spatial resolution of soft tissue information for tumor contour and localization, while the real-time and three-dimensional high spatial resolution of hard tissue information for tumor contour and localization could be provided by CT imaging. The use of noninvasive imaging like MRI and CT, possible with our MCAs, can provide the complementary information necessary for accurate evaluation of the tumorigenesis areas, which is one of the major challenges of diagnostic imaging.

## Results and Discussion

### Synthesis and Characterization of F-AuNC@Fe_3_O_4_

It have been demonstrated that the size control and further surface functionalization are two key factors in the development of high-performance nanoprobes for tumor targeting^13^. Because AuNC@Fe_3_O_4_ has no selectivity toward tissues and is unable to discriminate between malignant and nonmalignant tumors, conjugation of specific bimolecular (folic acid) with AuNC@Fe_3_O_4_ has been employed to improve their lesion targeting selectivity. The final nanoparticles were purified by gel filtration using Sephacryl HR-300 gel medium.

**Figure 2:**
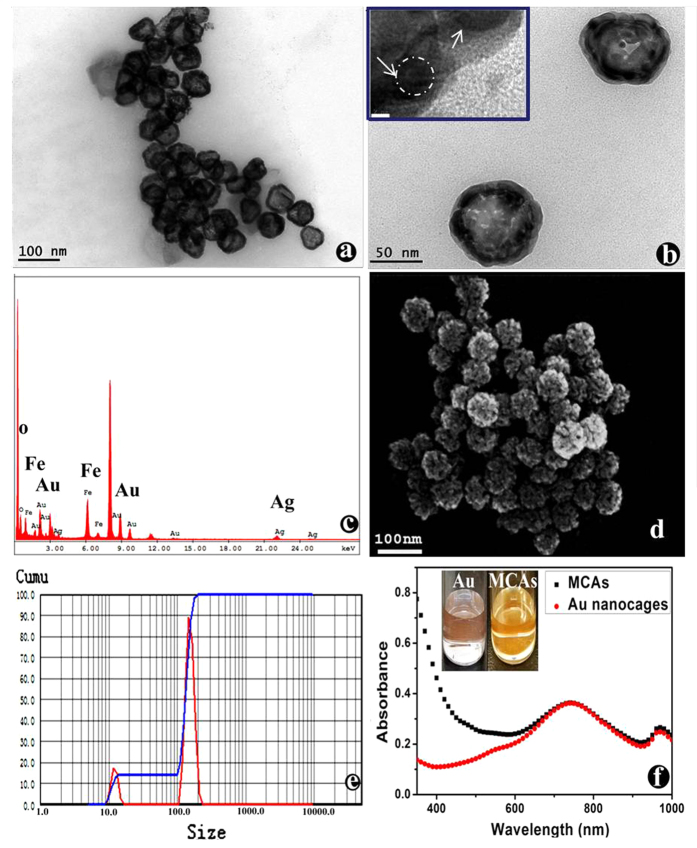
The characterizations of MCAs(F-AuNC@Fe_3_O_4_): TEM (**a,b**) (The inset is the High Resolution TEM); EDX (**c**); SEM (**d**); DLS (**e**) and UV-vis absorption of Au nanocages and MCAs (the inset is digital images under daylight).

High-resolution transmission electron microscopy (HRTEM) images of the ultra-small sized Fe_3_O_4_ nanoparticles and Au nanocage are shown in Figs S1 and S2 (Supporting Information). The average diameter of the Fe_3_O_4_ nanoparticles is around 2.2 nm with high uniformity, which could provide simultaneous *T*_*1*_ and *T*_*2*_ weight MRI contrast imaging proved in our previous work^9^. Au nanoparticles exhibit perfect nanocage structure with outer diameters of around 50 nm^14^. After assembled together, the HRTEM and SEM of final F-AuNC@Fe_3_O_4_ are shown in Fig. 2a,b,d, which clearly show that the prepared F-AuNC@Fe_3_O_4_ have hollow structure with rough morphology and they exhibit narrow distribution size around 70 nm. Compared with Au nanocages, it is obviously that the surface of F-AuNC@Fe_3_O_4_ was deposited with ultrasmall Fe_3_O_4_ nanoparticles. From the inset of Fig. 2b, the HRTEM of the edge of F-AuNC@Fe_3_O_4_, we can also clearly see that the small Fe_3_O_4_ nanoparticles around 2 nm are conjugated with Au nanocages. The energy-dispersive X-ray (EDX) spectrum of F-AuNC@Fe_3_O_4_ (Fig. 2c) also confirms the existence of elements O and Fe from Fe_3_O_4_, Au and Ag from the Au nanocages. The structure of F-AuNC@Fe_3_O_4_ can give enough space to let Fe_3_O_4_ nanoparticles connect with water molecular in tissue to produce high MRI signals due to the Fe_3_O_4_ nanoparticles assembled on the outer surface of Au nanocage; and at the other hand, the nanocages structure also gives their high surface to volume ratio, which also could benefit CT signal enhancement^15^,^16^. The Dynamic light scattering (DLS) (Fig. 2e) showed that the hydrodynamic diameter of F-AuNC@Fe_3_O_4_ was around 110 nm, which indicated that they have smaller size in aqueous without any aggregation and could process reasonable blood circulation time and good lesion accumulation rate^13^. Inductively coupled plasma mass spectrometry (ICP-MS) study revealed that weight percentages of Au and Fe_3_O_4_ encapsulated were 58.2 and 17.2%, respectively.

The UV-vis absorption spectra of the Au nanocage and F-AuNC@Fe_3_O_4_ in aqueous suspension are shown in Fig. 2f, and the absorption spectra of ultra-small sized Fe_3_O_4_ nanoparticles is provided in Fig. S3 (Supporting Information). Here, Au nanocages and F-AuNC@Fe_3_O_4_ exhibits very strong and broad absorption from 600 nm to 800 nm with the maximum absorption at around 750 nm. After deposition of ultra-small sized Fe_3_O_4_ nanoparticles onto the surface of Au nanocage, a new broad absorption band appears in the spectrum of F-AuNC@Fe_3_O_4_, which is contributed by the strong absorption of Fe_3_O_4_ nanoparticles in visible spectral region^17^.

### CT, Relaxometric and Biocompatible Properties

**Figure 3:**
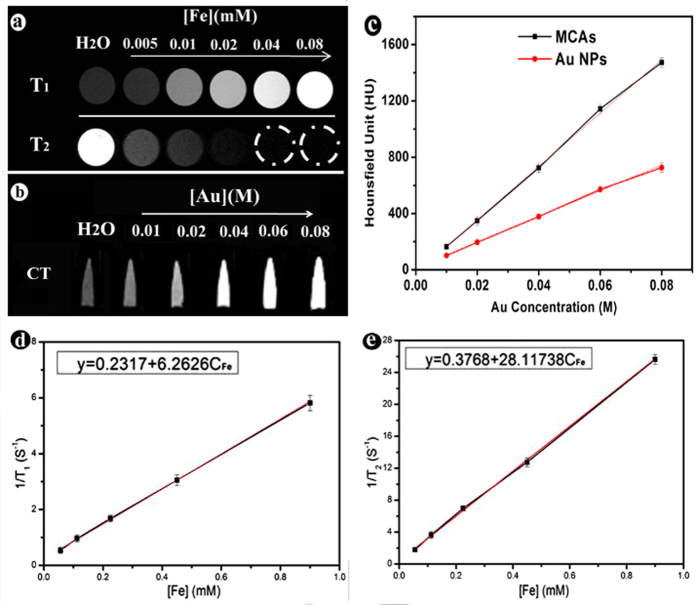
MRI and CT contrast ability of MCAs (F-AuNC@Fe_3_O_4_). (**a**) *T*_*1*_ *and T*_*2*_ weighted MR images of MCAs with different Fe ions concentrations. (**b**) CT imaging with different concentration of Au concentrations. (**c**) Plot of X-ray attenuation in Housfileld units (HU) as a function of Au concentration of MCAs (black squares), compared to pure gold nanoparticles (red circles). (**d,e**) Plot of *1/T*_*1*_ and *1/T*_*2*_ over Fe concentration of as synthesized MCAs.

CT and MRI images of F-AuNC@Fe_3_O_4_ nanoparticles at various concentrations in PBS solution were studied. F-AuNC@Fe_3_O_4_ possesses intrinsic advantages for X-ray CT imaging owing to the larger X-ray absorption efficiency of Au element. As known, the higher the atomic number and electron density is, the higher the attenuation coefficient is. Therefore, the developed F-AuNC@Fe_3_O_4_ was expected that have the good imaging ability in X-ray CT imaging. The X-ray attenuation (CT) potency of these F-AuNC@Fe_3_O_4_ with various concentrations of Au was examined and the pure Au nanoparticles (ca. 50 nm in diameter) were used as a control. It is clearly that the CT signal of F-AuNC@Fe_3_O_4_ increase in a concentration dependent manner (Fig. 3b,c). Interestingly, the F-AuNC@Fe_3_O_4_ exhibit higher X-ray attenuation potency than that of bare Au NPs at the same concentration. According to previous reports, smaller Au nanoparticles exhibited more pronounced X-ray attenuating capability than the larger one, which caused by the high surface to volume ratio and mainly contributed to X-ray attenuation^4^,^16^. Therefore, the enhanced CT contrast of F-AuNC@Fe_3_O_4_ compared to Au solid nanoparticle is attributed to their hollow and cage structure.

Moreover, we next evaluated the contrast capability of as prepared F-AuNC@Fe_3_O_4_ for *T*_*1*_ and *T*_*2*_ weight MRI at different Fe concentrations. The relaxation efficiencies are calculated by measuring the longitudinal and transverse relaxation rates (*r*_*1*_ and *r*_*2*_) of the proton signals of the F-AuNC@Fe_3_O_4_. As shown in Fig. 3d,e, F-AuNC@Fe_3_O_4_ exhibits a high *r*_*1*_ value of 6.263 and an *r*_*2*_ value of 28.117 mM^−1^ s^−1^. The high *r*_*1*_ relaxivity of F-AuNC@Fe_3_O_4_ all can be attributed to the large number 5 unpaired electrons of Fe^3+^ ions on the ultra-small Fe_3_O_4_ nanoparticle surface^5^. The *r*_*2*_*/r*_*1*_ ratio is also an important parameter to evaluate the efficiency of *T*_*1*_ contrast agents. The *r*_*2*_*/r*_*1*_ ratio of F-AuNC@Fe_3_O_4_ was 4.48 demonstrating that it can be efficient *T*_*1*_ contrast agents. Although there are other nanomaterials based on Fe_3_O_4_-Au for biomedical imaging^4^,^5^,^6^,^7^, as we know F-AuNC@Fe_3_O_4_ is the first example that exhibits high *r*_*1*_ relaxivity value for *T*_*1*_-weight MR enhanced imaging. Figure 3a also demonstrated the *T*_*1*_ and *T*_*2*_ modal MR images of F-AuNC@Fe_3_O_4_ with different Fe ion concentrations. The signal enhancement progressively increases with increasing of the F-AuNC@Fe_3_O_4_ concentrations, which manifests the potential of F-AuNC@Fe_3_O_4_ as powerful multifunctional contrast agent for *T*_*1*_ and *T*_*2*_ MRI.

For biomedical applications, it is necessary to guarantee the hemocompatibility and cytocompatibility^7^,^18^. Hemolytic assay and cell viability was used to assess the biocompatibility of the F-AuNC@Fe_3_O_4_, the results (Figs S4 and S5, Supporting Information) show synthesized F-AuNC@Fe_3_O_4_ have almost negligible damage to the red blood cells (<5% hemolytic activity) and have little toxicity to human lung cancer cell line A549 and normal human umbilical vein endothelial cells.

### *In vitro* cellular uptake assay

**Figure 4:**
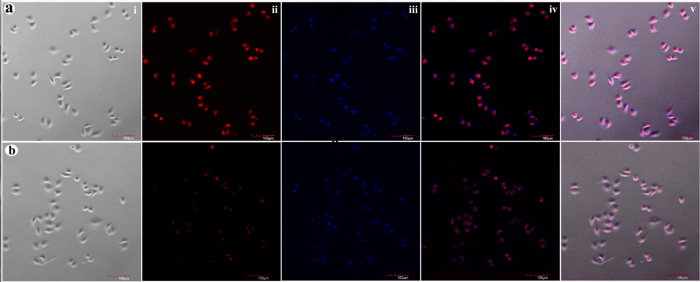
Confocal images of the A549 cells after one hour incubation with the 0.5 mg/mL of fluorescent F-AuNC@Fe_3_O_4_ (**a**); 0.5 mg/mL of fluorescent AuNC@Fe_3_O_4_ (**b**). The horizontal images were in this sequence: bright field (i), the red fluorescence field (ii), the nuclei stained by DAPI (iii), overlayer of DAPI and fluorescence (iv), overlayer of all (v). Scale bars are 100 μm.

The targeting ability of F-AuNC@Fe_3_O_4_ to folate receptor-overexpressed cancer cells was studied using A549 cells as an example. To more directly display the targeting capabilities of the F-AuNC@Fe_3_O_4_, the fluorescence Cy5-PEG-SH was selected to label the nanoparticls. In order to give clear fluorescence imaging and retain the ability of bio-functionalization, firstly, the mixture of COOH-PEG-SH and fluorescence Cy5-PEG-SH with 9:1 ratio was used to functionalize the surface of AuNC@Fe_3_O_4_, and then fluorescent AuNC@Fe_3_O_4_ was conjugated with targeting molecular folic acid to form the fluorescent F-AuNC@Fe_3_O_4_ for targeting cancer cell. As a control group, the fluorescent AuNC@Fe_3_O_4_ without surface folic acid also was used, which showed similar physical properties to those of fluorescent F-AuNC@Fe_3_O_4_. Figure 4a,b row show the confocal images of A549 cells after incubation with fluorescent F-AuNC@Fe_3_O_4_ and AuNC@Fe_3_O_4_ suspensions at a concentration of 0.5 mg/mL for one hour, respectively. The fluorescence image shown in Fig. 4a(i) clearly shows the successful internalization of the fluorescent F-AuNC@Fe_3_O_4_ into A549 cells. The much brighter red emission observed in Fig. 4a(i) as compared to that in Fig. 4b(i) (A549 cell incubation with fluorescent AuNC@Fe_3_O_4_) indicates that more F-AuNC@Fe_3_O_4_ can be internalized into A549 cells via folate receptor-mediated endocytosis, and the F-AuNC@Fe_3_O_4_ have more capability for cancer targeting recognition. These results confirm the F-AuNC@Fe_3_O_4_ can be effectively targeted to the folate receptor expressed cancer cells.

### *In vivo* CT, T_1_ and T_2_ modal MR Imaging

**Figure 5:**
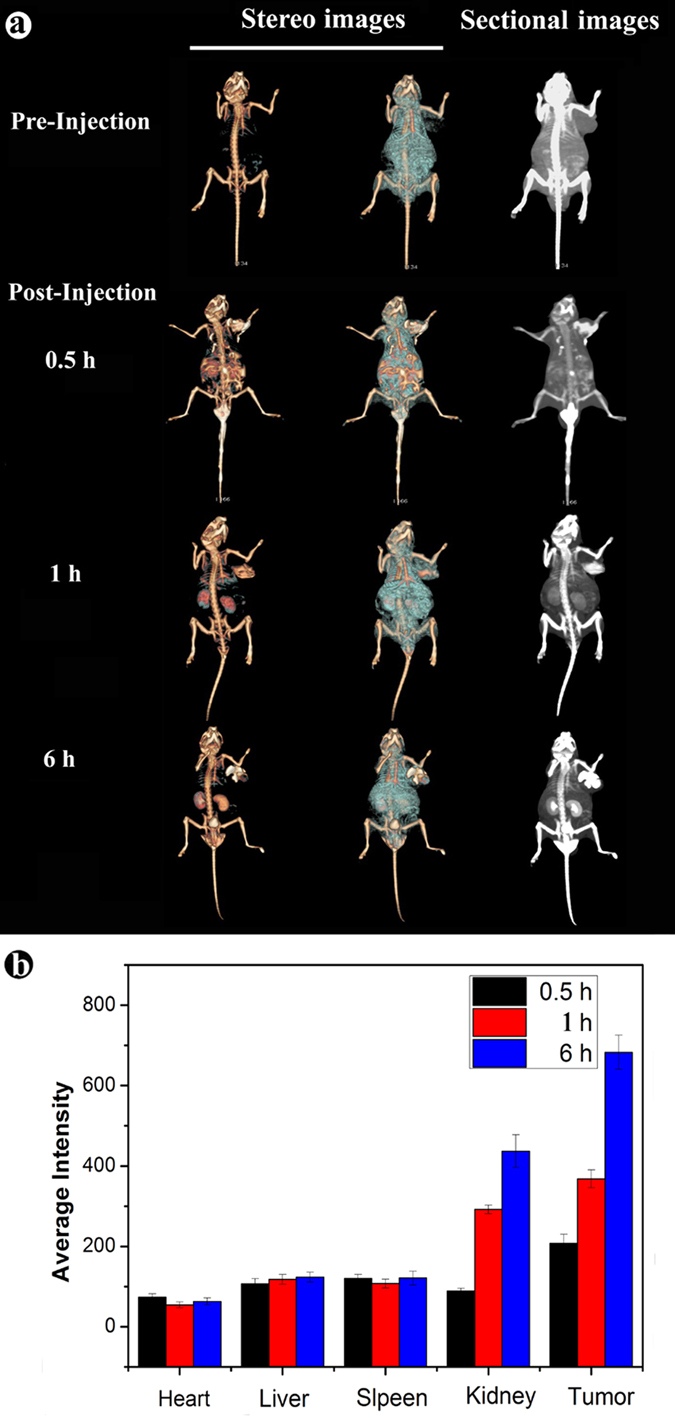
*In vivo* CT images of nude mice bearing tumor after intravenous injection of Au NPs at different timed intervals (pre-injection, 0.5 h, 1 h and 6 h post-injection), respectively (**a**) The HU average intensity of some organs (Heart, Liver, Spleen, Kidney, Tumor) after intravenous injection of Au NPs at different timed intervals.

For *in vivo* CT imaging, the CT imaging and CT value of the important organ regions were recorded before injection and at different time points post injection (Fig. 5a,b). Compared with the image of pre-injection, a great contrast enhancement was observed in the mouse body, tail vein and tumor region at 0.5 hour after injection, (Fig. 5a), demonstrating that the as synthesized F-AuNC@Fe_3_O_4_ can do enhance CT imaging in the circulating system. It is noted that the F-AuNC@Fe_3_O_4_ have a clear tendency of accumulation in the tumor tissue, the time-dependent distribution of the F-AuNC@Fe_3_O_4_ in the mouse was also tracked by CT signal value after intravenous injection. At timed intervals, the evident enhancement of the signals in different organs could be seen in Fig. 5b, the kidney and tumor imaging were greatly enhanced from 0.5 h to 6 h, and HU value of them rose from 95 to 464 and 213 to 730, respectively, while the signal value in other organs shows little fluctuation. A more careful look at the 3D-renderings of CT images, after post injection 6 h, the CT contrast intensity in the body and organs of mouse obviously decrease over time, while the region of the tumor tissue is still labeled and CT imaging of bladder organ are suddenly clear, showing the excellent tumor targeting and renal clearance properties of F-AuNC@Fe_3_O_4_. The slow elimination of F-AuNC@Fe_3_O_4_ from blood during circulation is attributed to their optimal particle size and surface functionalization. The results demonstrate that the F-AuNC@Fe_3_O_4_ have the feasibility to be served as an *in vivo* CT contrast agent to provide the real-time and 3D-high spatial resolution imaging.

**Figure 6:**
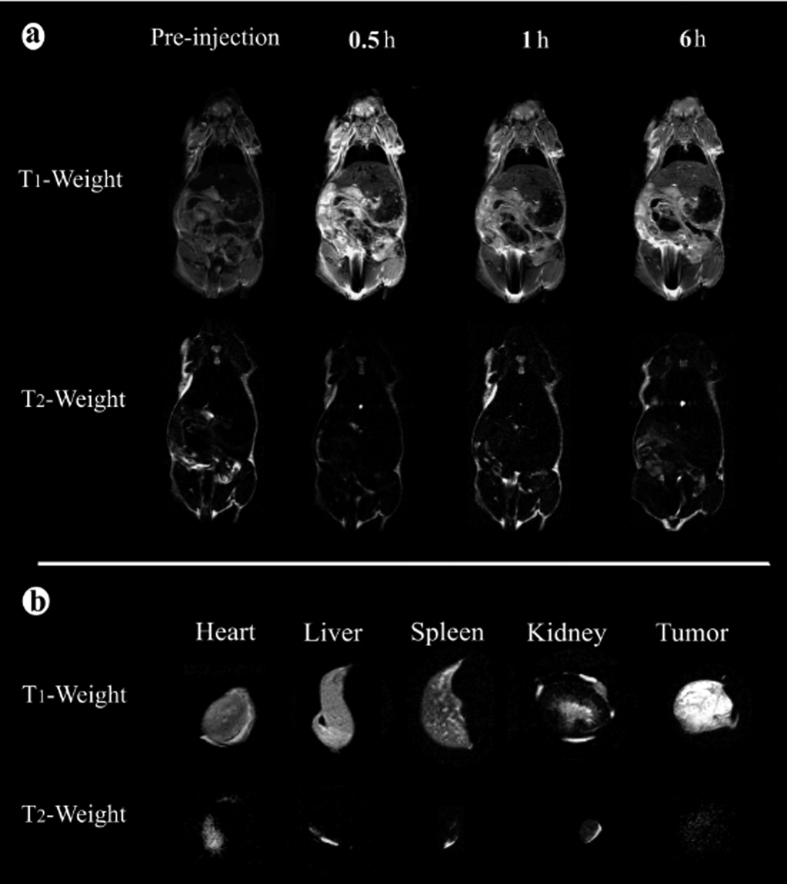
*In vivo T*_*1*_ and *T*_*2*_ weight MRI images of nude mice bearing tumor after intravenous injection of F-AuNC@Fe_3_O_4_ at different timed intervals (**a**); The *T*_*1*_ and *T*_*2*_ weighted MR images of various organs from the mice treated with F-AuNC@Fe_3_O_4_ after 6 h post-injection.

For tumor targeting *T*_*1*_ and *T*_*2*_ modal MR imaging, the F-AuNC@Fe_3_O_4_ with higher *r*_*1*_ and *r*_*2*_ relaxivity are expected to enhance the *T*_*1*_ and *T*_*2*_ modal MRI and to ease the toxicity with a decreased dose. *T*_*1*_ and *T*_*2*_ modal MRI of tumor-bearing mice were also recorded at various time intervals: before injection, 0.5, 1 and 6 hours after injection, as shown in Fig. 6a. Compared with pre-injection, we could observe a great contrast enhancement (brighten on the *T*_*1*_-weighted and darken on the *T*_*2*_ weighted MR images) in the mouse body after post injection, demonstrating that as synthesized F-AuNC@Fe_3_O_4_ can simultaneously enhance *T*_*1*_ and *T*_*2*_ relaxation in the circulating system. The enhanced signal of blood vessel can be maintained for more than 6 h, which is much longer than that of Gd complex small molecules with a high excretion rate (about several minutes in small animals)^9^,^19^. These results show that F-AuNC@Fe_3_O_4_ can also be used for long-term blood pool *T*_*1*_ and *T*_*2*_ modal MRI contrast agent, which is very important in clinical MR imaging^20^,^21^. The long-term effect is also critical to obtain high-resolution and steady state images. Similar with the CT imaging, after 6 h, the *T*_*1*_ and *T*_*2*_ contrast intensity in the body of mouse obviously decrease over time, while the contrast intensity in the region of tumor increased over time, which confirm the F-AuNC@Fe_3_O_4_ have a tendency to be enriched in the tumor tissue.

In general, once injected intravenously, the contrast agents are easily accumulated in liver and spleen tissues^22^. Therefore, to provide further support of tumor targeting ability as well as information on the biodistribution of the F-AuNC@Fe_3_O_4_, both tumor and important organs such as heart, liver, spleen, kidney and tumor were harvested after the *in vivo* experiments, and the MRI with *T*_*1*_ and *T*_*2*_ modal was shown in Fig. 6b, respectively. At first, the much higher *T*_*1*_ and *T*_*2*_ signals in the tumor tissue compared to those from other organs further confirm the specific targeting of the FMNPs to the tumor. And then, the imaging signal of organs might be difference between MRI imaging and CT imaging, which is why we integrate MR imaging with CT imaging for achieving their complementary imaging features.

### Histological study

**Figure 7:**
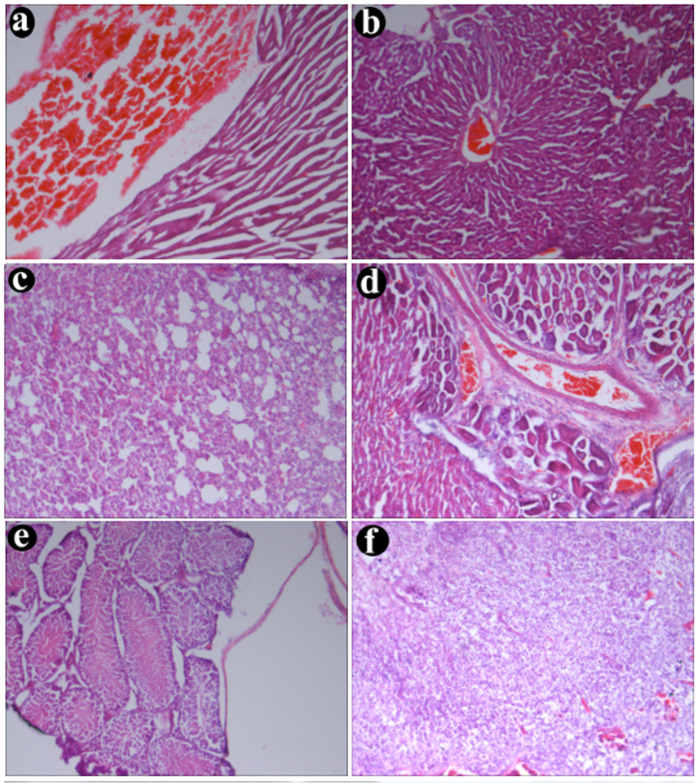
H&E staining of the organs sections (**a**) heart; (**b**) liver; (**c**) lung; (**d**) kidney; (**e**) bladder and (**f**) tumor) harvested from mice at 6 h post-injections.

An essential feature of MCAs is their biocompatibility, hence primary organs of tumor bearing mice post the administration of F-AuNC@Fe_3_O_4_ were tested via the pathological assay (H&E staining). In Fig. 7, the accumulation of F-AuNC@Fe_3_O_4_ was obviously observed in kidney, bladder and tumor (original magnification ×100), but it was little even absence in liver and heart at 6 h post the injection, which is in consistent with our *in vivo* imaging findings. In addition, no observable morphological changes were seen in these organs, which ruled out the presence of acute injury induced by the injection of F-AuNC@Fe_3_O_4_.

In summary, water-dispersible F-AuNC@Fe_3_O_4_ nanoparticles-based multimodal contrast agent has been developed for synergistically multimodal imaging of tumor. To the best of our knowledge, this is the first time that ultra-small sized Fe_3_O_4_ and Au nanocages were assembled together to provide simultaneous CT imaging and *T*_*1*_ and *T*_*2*_-modal MRI of tumor bearing mice. As a result, due to the small sizes and surface modification, the as-prepared F-AuNC@Fe_3_O_4_ preferentially accumulate in the tumor, and can be rapidly eliminated from the body via the renal system. The experimental results suggest the potential of F-AuNC@Fe_3_O_4_ as a safe and efficient contrast agent for biomedical applications such as multi-modality imaging and molecular diagnostics of diseases *in vitro* and *in vivo*, as well as theranostic applications.

## Methods

All experimental protocol including any relevant details were approved by the Regional Ethics Committee, Liaoning Medical University, Liaoning Province, China.

### Materials and Instrumentations

All of the chemicals and solvents were purchased from Sigma-Aldrich unless indicated otherwise. Transmission electron microscope (TEM) and energy dispersive X-ray (EDX) analyses were recorded using a FEI TECNAI G20 high-resolution transmission electron microscope operating at 200 kV. The samples were prepared by depositing a drop of a diluted colloidal solution on a carbon grid and allowing the liquid to dry in air at room temperature. Scanning electron microscope (SEM) data was collected by Hitachi Limited S4800. Dynamic light scattering (DLS) measurement was performed on a Malvern Zetasizer NANO ZS. UV-vis spectroscopy was carried out with SpectraMax^®^ M5 Microplate Reader using 96-well plate . The relaxation measurements were performed on a Bruker minispec mq60 NMR analyzer at 40 °C using a magnetic field of 1.41 T. Samples were diluted in MilliQ water to different Fe and Au concentrations in the approximate range, and the absolute concentrations were determined afterwards by ICP-MS.

### Synthesis of Gold Nanocages (AuNCs)

Herein, Au cages with hollow structures using Ag nanocubes as sacrificial templates have been prepared according to previous methods^12^. For all of the experiments, we used Au nanocages of mean 50 nm size together with a pore size of 2–3 nm. Briefly, 500 μL of the Ag nanocubes (3 nM) was added to 5 mL of deionized water containing poly (vinyl pyrrolidone) (PVP, 1 mg mL^−1^) hosted in a 50 mL flask under magnetic stirring and then heated to boil for 10 min. Then around 4 mL of aqueous solution of HAuCl_4_ (0.1 mM) was added to the flask at a rate of 0.75 mL min^−1^ until the solution had an optical extinction peak at 755 nm as confirmed by UV spectroscopy. The solution was refluxed for another 10 min until the color of the reaction was stable. Once cooled to room temperature, the sample was centrifuged and washed with saturated NaCl solution to remove AgCl and with water several times to remove PVP and NaCl. For the further experiment, The PVP was replaced on nanocages with 2-aminoethanethiol to obtain AuNC-NH_2_.

### Synthesis of AuNC@Fe_3_O_4_

In a typical experiment for the synthesis of 2.2 nm sized ultra-small Fe_3_O_4_ particles was prepared according to our previous experiment. First, the polymer poly(acrylic acid) (PAA, 5.56 mmol) was dissolved in Milli-Q water (50 mL) in a 100 mL three-necked flask bubbled with nitrogen air for 40 min to remove oxygen. Then the solution was heated up to 80 °C. Meanwhile, Ferric slat (0.54 mmol) and Ferrous salt (0.279 mmol) as iron precursors was quickly injected into the hot polymer solution under vigorous stirring in a nitrogen atmosphere, followed by drip addition of concentrated ammonia solution (15 mL, 28%) to adjust the pH value to 9–10. After refluxing for one hour, we can get the carboxyl group functionalized ultrasmall Fe_3_O_4_ particles.

The resultant carboxyl unit on the surface of Fe_3_O_4_ nanoparticels was activated by N-(3-Dimethylaminopropyl)-N–ethylcarbodiimide (EDC) and N-Hydroxysuccinimide (NHS), and subsequently treated with AuNC-NH_2_ to obtain AuNC@Fe_3_O_4_. 10 mg of excessive ultrasmall Fe_3_O_4_ nanoparticles was reacted with 10 mg NHS and 20 mg EDC in 5 mL of water, stirring at room temperature for 30 min before adding to the 5 mg of AuNC-NH_2_ suspension. Then the mixture was stirred at room temperature for another 24 h, followed by centrifugation and washing with ethanol and water, and then dispersed in 5 mL of ultra-purified water.

### Bio-functionalized of AuNC@Fe_3_O_4_ with folic acid to form the F-AuNC@Fe_3_O_4_

AuNC@Fe_3_O_4_ were first functionalized with COOH-PEG-SH and then conjugated with biomolecular for *in vitro* and *in vivo* experiments. Briefly, 5.0 mL of prepared AuNC@Fe_3_O_4_ water solution was added to 5.0 mg of COOH-PEG-SH and reacted overnight at 4 °C under stirring. The excess COOH-PEG-SH was removed by centrifugation at 12000 rpm for 8 min and washed five times with water to obtain PEGylated AuNC@Fe_3_O_4._ The PEGylated AuNC@Fe_3_O_4_ was dispersed in 0.1 M (pH 7.4) phosphate buffer to remove any trace metal.

The bio-conjugation was performed by EDC-catalyzed peptide bond formation between the carboxyl group on the particle surface and amino group on folic acid. First, 10 mL of freshly prepared EDC solution (10 mg mL^−1^ in weak acidic MES buffer solution) was mixed with 1 mL HNPs solution with stirring for one hour. Then, 50 mL folic acid solution (containing 0.4% NaOH) was added to the solution and mixed well on a vortex, and then the above mixture was left on a rotary shaker overnight at room temperature. After that, the Triton-X 100 (0.25% (w/v), 20 mL) and BSA (2% (w/v), 20 mL) were added. The mixture was then left on the rotary shaker for one hour. Finally, the resulting folic acid functionalized AuNC@Fe_3_O_4_ (F-AuNC@Fe_3_O_4_) were separated from free biomolecules by gel filtration using Sephacryl HR-300 gel medium.

### *In vitro* cytotoxicity study

Human lung adenocarcinoma A549 cells and human umbilical vein endothelial cells (HUVEC) were cultured in 25 cm^2^ flasks in Dulbecco’s Modified Eagle’s Medium DMEM (Gibco) containing 10% (v/v) fetal bovine serum (Gibco) at 37 °C in an atmosphere of 5% (v/v) CO_2_ in air. The media were changed every two days, and the cells were passaged by trypsinization before confluence. Before experiment, the cells were pre-cultured until confluence was reached. For studying the cytotoxicity, both A549 and HUVEC cells were seeded in a 96-well plate at a density 10^4^ cells/well for 24 h at 37 °C in 5% CO_2_. Then, the cells were treated with F-AuNC@Fe_3_O_4_ at different concentrations of 100, 200, 300, 400, 500 and even 1000 μg mL^−1^. After incubation for 24 h, cell viabilities were tested by standard MTT (3-(4,5)-dimethylthiahiazo-2-yl)-2,5diphenyltetrazolium bromide) assay. Cells incubated in the absence of nanoparticles were used as a control. All experiments were performed in triplicate.

### Hemolysis assay

In order to detect whether F-AuNC@Fe_3_O_4_ will cause damage to red blood cells (RBCs) after being injected into the blood vessels, a hemolysis assay experiment was carried out. The red blood cells were obtained by removing serum from the blood by centrifugation and suction. The 0.5 mL cells suspension was then mixed with: (a) 1 mL of PBS as a negative control; (b) 1 mL deionized water as a positive control; (c) 1 mL nanoparticle PBS solution with different concentrations of 100, 200, 400, 800 and 1000 μg mL^−1^. The mixtures were then vortexed for 2 h at room temperature. The samples were centrifuged and the absorbance spectrum of the supernatants was measured by UV-Vis characterization.

### *In vitro* targeting cellular imaging

For to evaluate targeting capabilities of the F-AuNC@Fe_3_O_4_, the fluorescence Cy5-PEG-SH was used to track the F-AuNC@Fe_3_O_4_. Firstly, the mixture of COOH-PEG-SH and fluorescence Cy5-PEG-SH with 9:1 ratio was used to functionalize the surface of AuNC@Fe_3_O_4_, and then fluorescence AuNC@Fe_3_O_4_ was conjugation with targeting molecular folic acid to form the fluorescence F-AuNC@Fe_3_O_4_ for targeting cancer cell. All of the modified procedures can be done as described above. In the next step, the A549 cells (1 × 10^5^ cells per well) were cultured in confocal imaging chambers (NET) at 37 °C for 24 h, then 200 μL of F-AuNC@Fe_3_O_4_ and AuNC@Fe_3_O_4_ (without bio-conjugation) with a concentration of 0.5 mg mL^−1^ were added to the chambers. After incubation for one hour, the cells were washed with a large amount of PBS to remove any free nanoparticles attached on the cell membrane and then stained with DAPI (10 μg mL^−1^) for 20 min, rinsed with PBS at least three times. And then, the Olympus-FluoView FV10 confocal laser-sca nning microscope was used to image morphology of the A549 cells and track the F-AuNC@Fe_3_O_4_ location in the A549 cells.

### *In vivo* CT, T_1_ and T_2_-weight MRI tri-modal imaging

Male BALB/c nude mice (4–6 weeks old) were purchased from the Cancer Institute & Hospital, Chinese Academy of Medical Sciences, Beijing. The Lung cancer A549 tumor-bearing models were generated by subcutaneous injection of 5 × 10^6^ cells in 50 μL of phosphate buffer solution (PBS) onto the left upper armpit of each mice. All the animal experiments were conducted in accordance with the guidelines of the Regional Ethics Committee, Liaoning Medical University, Liaoning Province, China. Firstly, the mice were anesthetized using 10% chloral hydrate (50 mL). Subsequently, 10 mg of F-AuNC@Fe_3_O_4_, dispersion solution was injected through the tail vein into the mouse. *In vivo* multi-imaging was performed at appropriate time points after tail vein injection.

CT imaging was acquired using Mediso nanoScan SPECT/CT produced by Mediso Ltd. Imaging parameters were as follows: slice thickness, medium; tube energy, 50 kVp, 670 μA; CTDIvol, 7279.9 cGy; semicircular parameters, full scan; Number of projections, 480; In-plane voxel size, medium. All animals were scanned in the cranial to caudal direction from the lower chest to the pelvis. CT data were analyzed using the Hounsfield units (HU) for regions.

*In vivo* MR imaging experiments were performed on a 3.0 T small animal MRI instrument (US Varian 3.0 T), and the pulse sequence used was a T_1_-weighted SE-XL/90 sequence with the following parameters: TR = 482 ms, TE = 16 ms: field of view [FOV]: 40 × 90 cm; matrix: 256 × 256; number of excitations (NEX): 5; slice thickness = 1 mm; FOV: 40 × 90 cm; coil: VOLUME; T2-weighted FSE-XL/90 sequence with the following parameters: TR = 3000 ms, TE = 48 ms: field of view [FOV]: 40 × 90 cm; matrix: 256 × 256; number of excitations (NEX): 5; slice thickness = 1 mm; FOV: 40 × 90 cm; coil: VOLUME.

### Biodistributionof F-AuNC@Fe_3_O_4_

To assess the specific *in vivo* tumor targeting of F-AuNC@Fe_3_O_4_, After injected with 10 mg F-AuNC@Fe_3_O_4_, the tumor bearing mice were sacrificed at 6 h post-administration and the tissues including heart, kidney, liver, spleen and tumor were harvested for isolated organ imaging.

### Histological study

Potential acute cytotoxicity and bio-distribution in major organs (heart, liver, lung, kidney, bladder and tumor) of tumor bearing mice post intravenous injection were assessed by dissection. Typical organs were harvested from the rats and immobilized in 4% paraformaldehyde at 4 °C for 48 h, and then embedded into paraffin. Sections from the organs were stained with hematoxylin and eosin (H&E) and observed under a light microscope at 100× magnification by an experienced physician, and representative images were provided.

## Additional Information

**How to cite this article**: Wang, G. *et al*. Au Nanocage Functionalized with Ultra-small Fe_3_O_4_ Nanoparticles for Targeting *T_1_*–*T2* Dual MRI and CT Imaging of Tumor. *Sci. Rep*. **6**, 28258; doi: 10.1038/srep28258 (2016).

## Supplementary Material

Supplementary Information

## Figures and Tables

**Figure 1:**
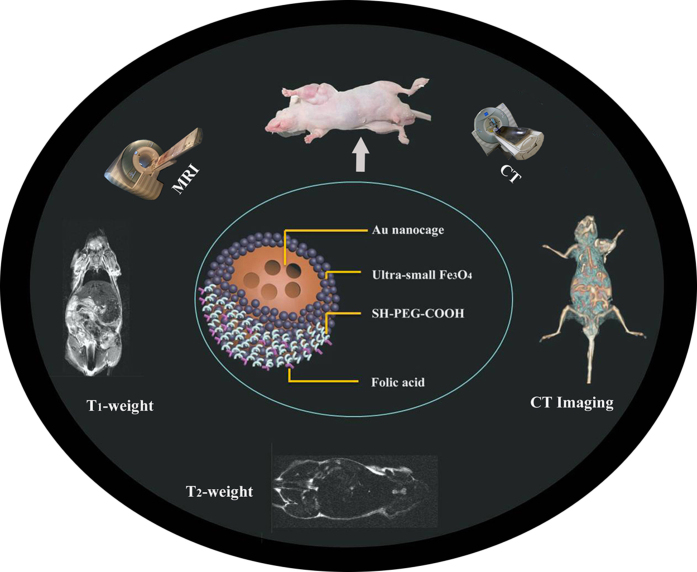
The illustration of MCAs (F-AuNC@Fe_3_O_4_) for targeting multimodal imaging of tumor.

## References

[b1] Chen W., Zhuang H., Cheng G., Torigian D. A. & Alavi A. Comparison of FDG-PET, MRI and CT for post radiofrequency ablation evaluation of hepatic tumors. Ann Nucl Med 27, 58–64 (2013). 23054830 10.1007/s12149-012-0656-6

[b2] Zhang Y. M., Jeon M., Rich L. J., Hong H., Geng J. M. . Non-invasive, multimodal functional imaging of the intestine with frozen micellar naphthalocyanines. Nat Nanotechnol 9, 631–638 (2014). 24997526 10.1038/nnano.2014.130 PMC4130353

[b3] Weissleder R. & Pittet M. J. Imaging in the era of molecular oncology. Nature 452, 580–589 (2008). 18385732 10.1038/nature06917 PMC2708079

[b4] Zhao H. Y., Liu S., He J., Pan C. C., Li H. . Synthesis and application of strawberry-like Fe3O4-Au nanoparticles as CT-MR dual-modality contrast agents in accurate detection of the progressive liver disease. Biomaterials 51, 194–207 (2015). 25771010 10.1016/j.biomaterials.2015.02.019

[b5] Huang J., Guo M., Ke H., Zong C., Ren B. . Rational design and synthesis of γFe_2_O_3_@Au magnetic gold nanoflowers for efficient cancer theranostics. Adv Mater 27, 5049–5056 (2015). 26198387 10.1002/adma.201501942

[b6] Amendola V., Scaramuzza S., Litti L., Meneghetti M., Zuccolotto G. . Magneto-plasmonic Au-Fe alloy nanoparticles designed for multimodal SERS-MRI-CT imaging. Small 10, 2476–2486 (2014). 24619736 10.1002/smll.201303372

[b7] Zhu J., Lu Y. J., Li Y. G., Jiang J., Cheng L. . Synthesis of Au–Fe3O4 heterostructured nanoparticles for *in vivo* computed tomography and magnetic resonance dual model imaging. Nanoscale 6, 199–202 (2014). 24241910 10.1039/c3nr04730j

[b8] Liu G., Gao J. H., Ai H. & Chen X. Y. Applications and potential toxicity of magnetic iron oxide nanoparticles. Small 9, 1533–1545 (2013). 23019129 10.1002/smll.201201531

[b9] Wang G. N., Zhang X. J., Skallberg A., Liu Y. X., Hu Z. J. . One-step synthesis of water-dispersible ultra-small Fe_3_O_4_ nanoparticles as contrast agents for T_1_ and T_2_ magnetic resonance imaging. Nanoscale 6, 2953–2963 (2014). 24480995 10.1039/c3nr05550g

[b10] He X. X., Liu F. Y., Liu L., Duan T. C., Zhang H. M. . Lectin-conjugated Fe2O3@Au core@shell nanoparticles as dual mode contrast agents for *in vivo* detection of tumor. Mol. Pharm. 11, 738–745 (2014). 24472046 10.1021/mp400456j

[b11] Li J. C., Hu Y., Yang J., Wei P., Sun W. J. . Hyaluronic acid-modified Fe_3_O_4_@Au core/shell nanostars for multimodal imaging and photothermal therapy of tumors. Biomaterials 38, 10–21 (2015). 25457979 10.1016/j.biomaterials.2014.10.065

[b12] Skrabalak S. E., Au L., Li X. D. & Xia Y. N. Facile synthesis of Ag nanocubes and Au nanocages. Nature Protocols 2, 2182–2190 (2007). 17853874 10.1038/nprot.2007.326

[b13] Bao F., Yao J. L. & Gu R. A. Synthesis of magnetic Fe_2_O_3_/Au core/shell nanoparticles for bioseparation and immunoassay based on surface-enhanced raman spectroscopy. Langmuir 25, 10782–10787 (2009). 19552373 10.1021/la901337r

[b14] Shi P., Li M., Ren J. S. & Qu X. G. Gold Nanocage-based dual responsive “caged metal chelator” release system: noninvasive remote control with near infrared for potential treatment of alzheimer’s disease. Advanced Functional Materials 43, 5412–5419 (2013).

[b15] Xie J. P., Zhang Q. B., Lee J. Y. & Wang D. I. C. The synthesis of SERS-active gold nanoflower tags for *in vivo* applications. ACS Nano 2, 2473–2480 (2008). 19206281 10.1021/nn800442q

[b16] Cole L. E., Ross R. D., Tilley J. M., Vargo-Gogola T. & Roeder R. K. Gold nanoparticles as contrast agents in x-ray imaging and computed tomography. Nanomedicine 10, 321–341 (2015). 25600973 10.2217/nnm.14.171

[b17] Sathe T. R., Agrawal A. & Nie S. M. Mesoporous silica beads embedded with semiconductor quantum dots and iron oxide nanocrystals: dual-function microcarriers for optical encoding and magnetic separation. Anal Chem 78, 5627–5632 (2006). 16906704 10.1021/ac0610309

[b18] Xiao Q., Bu W., Ren Q., Zhang S., Xing H. . Radiopaque fluorescence-transparent TaO_x_ decorated upconversion nanophosphors for *in vivo* CT/MR/UCL trimodal imaging. Biomaterials 33, 7530–7539 (2012). 22840224 10.1016/j.biomaterials.2012.06.028

[b19] Feng Y., Zong Y. D., Ke T. Y., Jeong E. K., Parker D. L. . Pharmacokinetics, biodistribution and contrast enhanced MR blood pool imaging of Gd-DTPA cystine copolymers and Gd-DTPA cystine diethyl ester copolymers in a rat mode. Model. Pharm. Res. 23, 1736–1742 (2006). 16850267 10.1007/s11095-006-9028-z

[b20] Tombach B., Reimer P., Bremer C., Allkemper T., Engelhardt M. . First-pass and equilibrium-MRA of the aortoiliac region with a superparamagnetic iron oxide blood pool MR contrast agent (SHU 555C): results of a human pilot study. NMR Biomed. 17, 500–506 (2004). 15523717 10.1002/nbm.906

[b21] Bjørnerud A. & Johansson L. The utility of superparamagnetic contrast agents in MRI: theoretical consideration and applications in the cardiovascular system. NMR Biomed. 17, 465–477 (2004). 15526351 10.1002/nbm.904

[b22] Linderoth S., Hendriksen P. V., Bødker F., Wells S., Davies K. . On spin-canting in maghemite particles. J Appl. Phys. 75, 6583–6585 (1994).

