# Supplementary material for: Au Nanocage Functionalized with Ultra-small Fe3O4 Nanoparticles for Targeting T1–T2Dual MRI and CT Imaging of Tumor
Source: Sci Rep. 2016 Jun 17;6:28258. doi: 10.1038/srep28258 (PMC4911575; doi:10.1038/srep28258)
Supplement: Supplementary Information [file srep28258-s1.doc]

Au nanocage functionalized with ultra-small Fe_3_O_4_ nanoparticles for targeting *T_1_* -*T_2_* dual MRI and CT imaging of tumor

Guannan Wang,^ab*^ Wei Gao,^a^ Xuanjun Zhang,^b*^ Xifan Mei^*a^

^a^ *College of Pharmacy & the Key Laboratory for Medical Tissue Engineering of Liaoning Province, Liaoning Medical University, Jinzhou, 121001, China. E-mail: chemwangguannan@gmali.com (Dr. Guannan Wang); meixifan1971@163.com (prof. Xifan Mei);*

^b^*Faculty of Health Sciences, University of Macau, Avenida da Universidade, Taipa, Macau, China.*


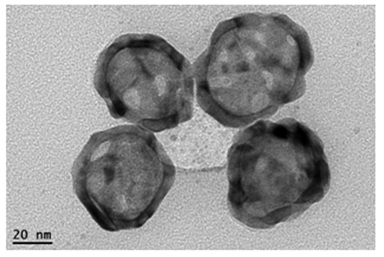


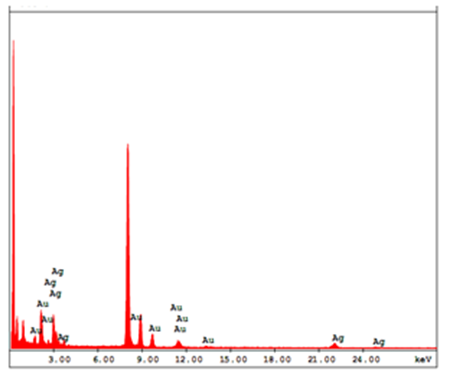


**Figure S1.** The HRTEM and EDX of Au nanocages


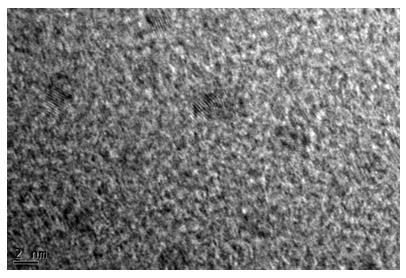


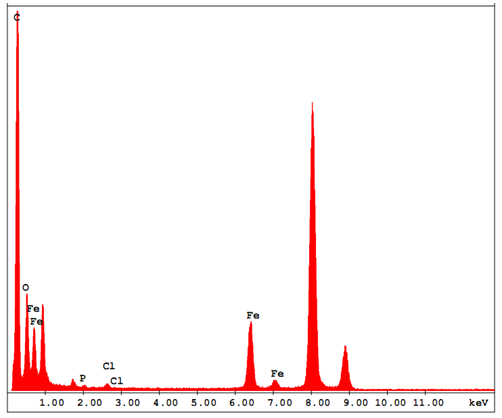


**Figure S2.** The HRTEM and EDX of ultra-small Fe_3_O_4_ nanoparticles


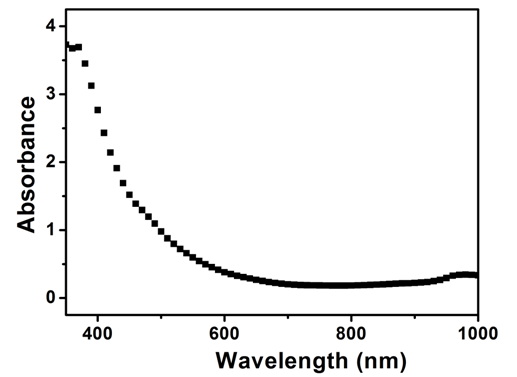


**Figure S3.** The UV absorption spectrum of ultra-small Fe_3_O_4_ nanoparticles

***Biocompatible of the as-Synthesized*** ***F-AuNC@Fe_3_O_4_*.**

For biomedical applications, it is necessary to guarantee the hemocompatibility and cytocompatibility. Hemolytic assay was used to assess the hemocompatibility of the F-AuNC@Fe_3_O_4_ based on the previous report. As shown in the Figure S4,. We can see that the F-AuNC@Fe_3_O_4_ at concentration ranging from 100 to 1000μg mL^-1^ do not cause any obvious hemolysis effect when compared with the negative control (PBS). In contrast, the positive control of water induces a signiﬁcant hemolysis of HRBCs. The exact RBC hemolyzed ratio was determined by measuring the absorbance of supernatants at 541 nm (hemoglobin) by UV-visible spectroscopy, the hemolysis percentages of HRBCs in the presence of F-AuNC@Fe_3_O_4_ at various concentrations were calculated to be 0.56%, 0.82%, 1.02%, 1.49%, and 2.81%, respectively. All of them are less than the threshold value of 5% hemolytic activity, which suggests that the presented F-AuNC@Fe_3_O_4_ have a good hemocompatibility and have almost negligible damage to the red blood cells.

The cytocompatibility of the F-AuNC@Fe_3_O_4_ was evaluated by cell viability assay using the human lung cancer cell line A549 and normal human umbilical vein endothelial cells (HUVEC) treated with the F-AuNC@Fe_3_O_4_. Figure S5 shows cell viability after incubated with F-AuNC@ Fe_3_O_4_ at different concentrations (100~100 μg mL^-1^) for 4 hour. For the MTT assay, the optical absorbance of formazan (produced by the cleavage of MTT by dehydrogenases in living cells) at a wavelength of 490 nm is directly proportional to the number of live cells. As a model for normal tissue cells, the experimental results of HUVEC show that more than 90% of the cell survived after 4 h of incubation, even at a high FMNPs concentration (1000 μg/mL). These results demonstrate F-AuNC@Fe_3_O_4_ have excellent and little toxicity of our nanoparticles as dual contrast agents.

Taken together with the results from hemolytic assay, we can safely conclude that the developed F-AuNC@ Fe_3_O_4_ has a good biocompatibility in the studied concentration range, which is essential for their further biomedical applications.


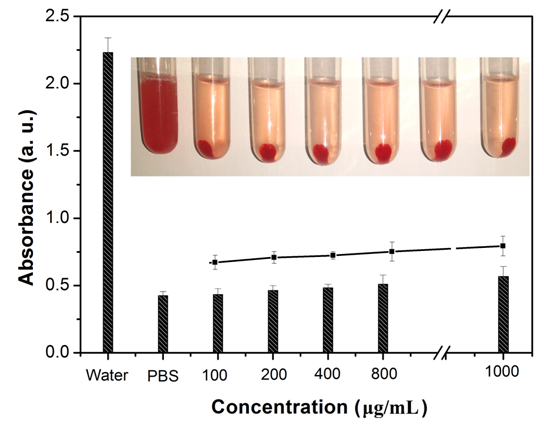


**Figure S4**. Hemolysis assay of F-AuNC@Fe_3_O_4_ by incubating RBCs with different concentrations.


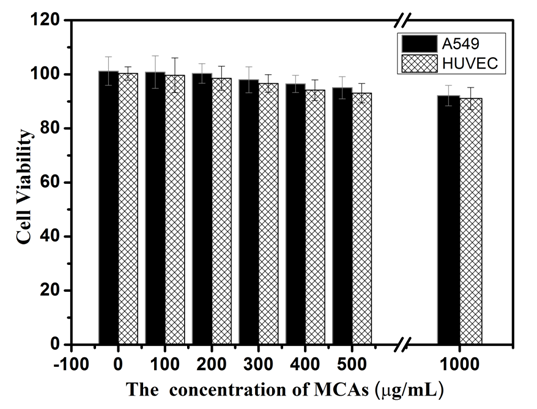


**Figure S5**. Cell viability of A549 and Human umbilical vein endothelial cells incubation with different concentration of F-AuNC@Fe_3_O_4_ for 4h.
